# The Role of Leadership in Public Sector Innovation: A Systematic Review and Meta-Analysis of the Management of COVID-19 in Asian Countries

**DOI:** 10.3389/fpubh.2021.743748

**Published:** 2021-12-15

**Authors:** Faizus Sazzad, V. Priya Rajan, Mehmet Akif Demircioglu

**Affiliations:** ^1^Department of Public Policy, Lee Kuan Yew School of Public Policy, National University of Singapore, Singapore, Singapore; ^2^Department of Surgery, Yong Loo Lin School of Medicine, National University of Singapore, Singapore, Singapore; ^3^Faculty of Arts and Social Science, National University of Singapore, Singapore, Singapore

**Keywords:** coronavirus, COVID-19, pandemic, leadership, public sector, innovation, policy

## Abstract

Strong leadership in public sector innovation can empower governments to address community challenges in new ways in light of the challenges posed by the global coronavirus pandemic. Coronavirus management policy, pandemic responses, needs, and options are reflected in various Asian countries in respective published literature, but a summarized synthesis is not available. Using a systematic review approach (PRISMA), this study has analyzed the role of leadership in public sector innovation in COVID-19 management and synthesized 23 articles from 23 different Asian countries. In the light of available data, public sector innovation (PSI) and the role played by the leadership of each country' have been found to be largely inter-dependent. The current review provides a cross-section of the ongoing nature of the pandemic, as management responses and trend data in the countries are still emerging or evolving. Additionally, our study contributes a current state report regarding the barriers facing the leadership of Asian countries in mitigating the global pandemic through PSI. Our study found that a strong political leadership presence combined with a technocratic approach and a highly-skilled public sector workforce, could lead to more tremendous success in managing the outbreak. Furthermore, religious leadership was also found to have a potentially significant role in COVID-19 management strategies.

## Introduction

Asia was the first region to be affected by the global pandemic of the Coronavirus (COVID-19), particularly countries in East and Southeast Asia which saw rapid growth in the number of confirmed cases and deaths when COVID-19 broke out in early 2020. The ensuing economic downturn caused by global trade disruptions, cessation of tourism, and regional containment measures brought on socio-economic challenges, which eventually forced some countries to take the initiative and adopt innovative measures in their overall policy responses for COVID-19 management (1, 2).

The purpose of this article is to examine the role of leadership in public sector innovation (PSI), specifically in addressing the challenges in public health management of the global COVID-19 outbreak from 2020 using a systematic review. This evaluation is based on a systemic review of scholarly studies of the leadership role in the various PSI adopted in 23 Asian countries to manage their respective COVID-19 outbreaks. This article provides a “state-of-art” summary of the PSI impact, categorized leadership role, and policy responses employed in Asian countries during the global pandemic.

PSI can be defined as novel services, technologies, organizational structures, management approaches, processes, or policies adopted by government agencies to address specific challenges confronting the organization, nation, or society (3–5). Leadership has been acknowledged as one of the most important factors influencing the success or failure of PSI (6, 7). Leadership in PSI can take the form of top-down innovation initiated by executive leadership and bottom-up innovation started by civil servants on the ground (8, 9). Particularly in a significant or national crisis, PSI tends to be “led by politicians” in response to crises (10). The COVID-19 pandemic is a prime example of a national crisis, and evaluating the management responses across different countries presents an opportunity to understand how effective leadership can drive PSI. This review explores these challenges for leaders and decision-makers and evaluates the generalizability of the leadership role in PSI processes across Asian nations.

The rest of the article is organized as follows: The first section will explain the rationale and context of the research. Section methods will provide information about methods, such as search strategy, which is a systematic review based on the Preferred Reporting Items for Systematic Reviews and Meta-analyses (PRISMA) method. Section results will report results, and section discussion will discuss the findings along with implications, limitations, and future research directions. Finally, this article ends with a conclusion. The main finding of this article is that leadership styles can influence the level of innovation adopted and the success or failure of the strategic response. More research in this area, particularly on religious leadership as a source of innovation, could be helpful. The statistical calculation may not reflect the impact of PSI and leadership role in the short term, and long-term impact is yet to be achieved, as the COVID-19 situation is continuously evolving.

### Rationale and Context of the Research

Much has been written since 2020 about the responses to COVID-19 across different countries (1, 2, 11). The novelty of the virus presented unknown and evolving challenges that continue to test the effectiveness of governments across the world in adopting containment and management strategies (11). Beyond the public health perspective, the national responses can thus offer insight into how top-down leadership (e.g., politicians, political appointees, or senior-level management) can directly influence the effectiveness of public administration responses. Although country cases and small-scale comparisons between a few countries have been published (12, 13), much of the literature published to date has focused on individual country-based studies, or small-scale studies involving a few selected countries, as mentioned above.

This current state of the literature could be attributed to the recency of the COVID-19 outbreak, as well as the continually evolving responses as countries grappled with unprecedented demand on their processes and problem-solving capabilities. Hence, this article's contribution to the current literature offers a systematic review of the government responses to COVID-19 management across countries. In addition, the value of systematic research has been noted as it provides reproducible, transparent, and standardized techniques to identify pertinent studies (14, 15).

The systematic review in this article focuses on scholarly publications analyzing leadership roles in PSI within Asia specifically. The two factors underpinning this rationale are the geographical point of origin of COVID-19 and the region's relative level of preparedness. Firstly, as Asia was the origin of the outbreak, the first transmissions within and beyond national borders were recorded in Asia (16). As such, Asian nations were among the first to have their governance and administrative capacity tested. Taiwan and Hong Kong, for example, were the first to send fact-finding missions to Wuhan in China to understand the potential public health risk. Not surprisingly, the first confirmed transmissions beyond China's borders were recorded in Asian countries, namely Thailand and Japan (17).

Therefore, it stands to reason that Asian governments were among the first nations to design, adopt, or refine their administrations' responses to address the early outbreak. Secondly, while all countries had to grapple with the unprecedented scale and nature of the COVID-19 epidemic, those in Asia had some prior experience in dealing with a broadly similar outbreak about 18 years earlier, during the SARS outbreak in 2003 (18). At that time, countries outside Asia had limited or no exposure and minimal cases. However, Asian countries, especially those in East Asia, had a higher degree of exposure, both in the number of cases and deaths. As such, the governments in Asia had put in place more processes in readiness for a potential new outbreak in the future. A systematic review of the studies on Asian governments' responses in harnessing PSI through leadership would thus be able to deepen the current understanding of how leadership can drive adequate PSI in managing this COVID-19 challenge.

Traditional approaches to the typology of leadership as a source of innovation within an organization present two broad types: bottom-up vs. top-down innovations (9). Bottom-up innovations typically arise from employees or work units at lower rungs of public sector organizations as they seek to improve aspects of their work processes. By contrast, top-down innovations are driven by politicians, political appointees, or senior-level management. In addition, they are “often associated with changes of government, new mandates or large-scale initiatives, and can involve a combination of new policy goals and frameworks that are sometimes associated with contemporary ideologies, as well as new concepts of services and service delivery” (p. 794) (19). The context always matters for the effects of leadership on PSI (9, 19, 20).

In the case of COVID-19, the nature and scale of the crisis mean that top-down leadership to drive innovation appears imperative. Both leadership and innovation are urgently required public sector-wide, within a short span of time. However, in a VUCA world (Volatile, Uncertain, Complex, Ambiguous) (20), the binary opposites of top-down vs. bottom-up leadership to drive innovation may be less applicable. Instead, the concept of horizontal leadership has been proposed (21) as a more appropriate response to innovation and problem-solving. Ansell et al. suggest that when dealing with such turbulent problems (i.e., VUCA problems) as the COVID-19 crisis, leaders need to drive “horizontal collaboration between professional groups and sectors, allowing the situation or task to set the team” (21) and lead people for whom there is “no formal leadership responsibility” [ibid]. Beyond seeking expert advice, leaders driving innovation to deal with VUCA problems will have to “accept cognitive dissonance and imperfect solutions, build alliances, learn from experience, adapt to new circumstances, and look for next practice” instead of seeking a “non-existing best practice” (pp. 955–956) (21).

The systematic review and the meta-analysis illustrates the feasible relationships among PSI and leadership in COVID-19 management. To affirm these relationships, published literature has been reviewed to get the potential answers to the following questions:

*R.Q. 1:* Was PSI adopted to manage COVID-19 in Asian countries?

*R.Q. 2:* Did leadership have an impact on nations' COVID-19 management strategies and approaches? If so, how?

The influence of leadership on the COVID-19 management was identified in Asian countries' policy responses from published articles in business forums (22). The findings from these articles and published literature have led to the presumption that PSI has been adopted in some Asian countries to mitigate the adverse effects of COVID-19.

## Methods

### Search Strategy

A systematic review was conducted according to the Preferred Reporting Items for Systematic Reviews and Meta-analyses (PRISMA) standard (23). We conducted electronic searches on Embase, Web of Science, Medline (via PubMed) database records from the date of inception to 31st May 2021. A repetitive and exhaustive combination of the “Emtree Headings” were used at Embase database, Web of Science database, and PubMed database with MeSH headings. An additional search for scholarly articles in Google Scholar and selective search to Public administration journals via Wiley Online Library, SAGE journals, and Emerald publishing journals were performed to authenticate the primary search.

A repetitive and exhaustive combination of the following “Emtree Headings” were used at Embase database: “public health service” /exp AND (“coronavirus disease 2019” /exp OR “2019 novel coronavirus disease” OR “2019 novel coronavirus epidemic” OR “2019 novel coronavirus infection” OR “2019-ncov disease” OR “2019-ncov infection” OR “covid' OR “covid 19” OR “covid 19 induced pneumonia” OR “covid 2019” OR “covid-10” OR “covid-19” OR “covid-19 induced pneumonia” OR “covid-19 pneumonia” OR “covid19” OR “sars coronavirus 2 infection” OR “sars coronavirus 2 pneumonia” OR “sars-cov-2 disease” OR “sars-cov-2 infection” OR “sars-cov-2 pneumonia” OR “sars-cov2 disease” OR “sars-cov2 infection” OR “sarscov2 disease” OR “sarscov2 infection” OR “wuhan coronavirus disease” OR “wuhan coronavirus infection” OR “coronavirus disease 2” OR “coronavirus disease 2010” OR “coronavirus disease 2019” OR “coronavirus disease 2019 pneumonia” OR “coronavirus disease-19” OR “coronavirus infection 2019” OR “ncov 2019 disease” OR “ncov 2019 infection” OR “new coronavirus pneumonia” OR “novel coronavirus 2019 disease” OR “novel coronavirus 2019 infection” OR “novel coronavirus disease 2019” OR “novel coronavirus infected pneumonia” OR “novel coronavirus infection 2019” OR “novel coronavirus pneumonia” OR “paucisymptomatic coronavirus disease 2019” OR “severe acute respiratory syndrome 2” OR “severe acute respiratory syndrome 2 pneumonia” OR “severe acute respiratory syndrome cov-2 infection” OR “severe acute respiratory syndrome coronavirus 2 infection” OR “severe acute respiratory syndrome coronavirus 2019 infection”).

The Web of science database searched for “COVID” AND “Leadership” in public service with all linked databased namely Web of science core collection, BIOSIS previewes, Current contents connects, KCI-Korean Journal database, Russian science citation index, SciELO citation index and Zoological records. On the PubMed database, a repetitive and exhaustive combination of the following “Medical Subject Headings” (MeSH) search terms were used: (“Leadership”[Majr]) AND (“COVID-19”[Mesh] AND “SARS-CoV-2”[Mesh]). Additional search for scholarly articles in Google Scholar and selective search to Public administration journals via Wiley Online library, SAGE journals and Emerald publishing journals were performed to authenticate the primary search.

### Enrollment Criteria

The unit of analysis in this study is at the country or national government level. We have included the published articles mentioning the government's role in COVID-19 pandemic management only if published in English. The role of leadership was a subject to ascertain via “topic,” “title,” and “abstract” review during the enrolment process. The inclusion criteria included the reviews of COVID-19 management and were restricted to Asian countries and regions. As Asia was the center of this disaster's first-situation-report, it is rational to investigate the Asian countries' responses to mitigate/control the large-scale effects. Additionally, multiple articles from the same country or a province of a country have been excluded as they would not reflect different leadership roles in the same population and may produce “synergistic inclusion” bias, and may lead to a “confounding” effect. National policy statements/papers were also excluded as they depict large-scale management plans of the particular country and remain beyond the scope of judgment due to a lack of comparative outcome analysis. It is also not unexpected to have government expressions of hopeful aspirations in the policy statement that may not be materialized in reality and which we believe in many cases were superseded anyway by the uncontrollable COVID-19 destructive effects.

Similarly, policy reports published by different government authorities were excluded for two reasons; firstly, these reports contain many domains beyond the scope of analysis of this review. Secondly, not all countries have published their COVID-19 policy reports, and they may have reflected the government achievements only. The smaller-scale case comparisons, especially between two nations or a few, remain out of the inclusion in this article, as these papers have already described a comparison. Hence they may induce directive business and have a confounding effect if added. However, we took the opportunity to review those papers and have cited them in this article whenever relevant. Media reports, critic papers, opinions, and reviews published in “online domains” were excluded from this manuscript as the validity and authenticity of the data could not be ascertained. Furthermore, for studies published from the same country or region, only those with more recent data were included. “Innovation” in this domain was not an active search criterion set by the authors, rather a fruitful concomitant outcome. We have included all public sector innovations implemented for COVID-19 management from an Asian perspective.

### Study Selection

The extracted citations were screened and assessed by the authors using reference manager software EndNote X9 independently for inclusion. The articles were first screened by their titles and abstracts, where criteria were purposely broad to include all relevant studies. Second stage review for studies that have made it through the first stage, or cases where a decision cannot be made, full-text reviews were performed on articles to confirm the relevance. To ascertain the conformity of the included studies, expert opinion was sought from one university in Asia. The final inclusion was guided, and an additional search was made to improve the sensitivity, where we have used citation chasing in Google Scholar and public administration publishing houses (via online). Further data was sought by manual search using the backward snowballing method.

### Quality of Evidence (QoE)

All the included studies were observational studies, with the majority reporting the leadership role of the respective countries in managing the COVID-19 disaster. As illustrated in chapter 11 of the Cochrane handbook of reviews (24), GRADEpro was used to evaluate the quality of evidence in the included studies (Table 1). In addition, all included studies were assessed for the specific outcome relating to the leadership role in COVID-19 management, namely, availability of an effective public sector administration of the country, formation of a national committee, crisis denial at early stage, digital surveillance, public sector innovation (PSI), community awareness level and role of scientific/physician's recommendation for disaster management.

**Table 1:** Assessment of the quality of the included evidence.

**Certainty assessment**							**No of patients**		**Effect**		**Quality of evidence**	**Importance**
**No of studies**	**Study design**	**Risk of bias**	**Inconsistency**	**Indirectness**	**Imprecision**	**Other considerations**	**The role of leadership**	**Public sector innovation**	**Relative (95% CI)**	**Absolute (95% CI)**		
**Effective public sector administration**												
23	Observational studies	Not serious	Not serious	Not serious	Serious^a^	None	19/23 (82.6%)	-	-	-	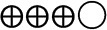 MODERATE	CRITICAL
**National Committee for COVID-19**												
21	Observational studies	Not serious^a^	Not serious	Serious	Serious^a^	None	17/19 (89.5%)	-	-	-	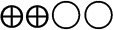 LOW	IMPORTANT
**Crisis denial at early stage**												
22	Observational studies	Serious^b^	Not serious	Not serious	Serious^a^	None	6/22 (27.3%)	-	-	-	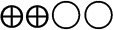 LOW	CRITICAL
**Digital surveillance**												
19	Observational studies	Not serious	Not serious	Serious^b^	Not serious	None	14/19 (73.7%)	-	-	-	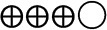 MODERATE	CRITICAL
**Public Sector Innovation (PSI)**												
23	Observational studies	Not serious	Serious^b^	Not serious	Not serious	None	8/23 (34.8%)	-	-	-	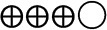 MODERATE	CRITICAL
**Community awareness level**												
23	Observational studies	Not serious	Not serious	Serious^b^	Serious^a^	None	18/23 (78.3%)	-	-	-	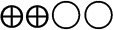 LOW	CRITICAL
**Scientists/physician's recommendation**												
18	Observational studies	Not serious	Not serious	Not serious	Serious^b^	None	13/18 (72.2%)	-	-	-	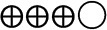 MODERATE	CRITICAL

*CI: Confidence interval*.

a *Not accessed*.

b *Uncertain*.

### Data Abstraction and Analysis

Authors abstracted details of the study characteristics, article information, essential policy information, leadership role assessment, and outcome and impact of COVID-19. Additionally, PSI in different countries was ascertained with their impact in diagnosing confirmed COVID-19 cases, number of death due to COVID-19, and vaccinated population till date were measured. Finally, the forest plots were generated utilizing the Review Manager 5 software (RevMan 5.4) (25).

Depending on the nature of the outcomes extracted from the journals, they were categorized either under dichotomous or continuous data type to generate effect measures in the form of risk ratio (RR) and mean difference (MD), respectively. The effect measures were calculated using the inverse variance method. The data were pooled into either a “random effects” or “fixed effects” model based on the *I*^2^ value. When there was high heterogeneity (*I*^2^ > 75%), a random effect model was utilized to account for statistical variability across studies.

## Results

**Figure 1:**
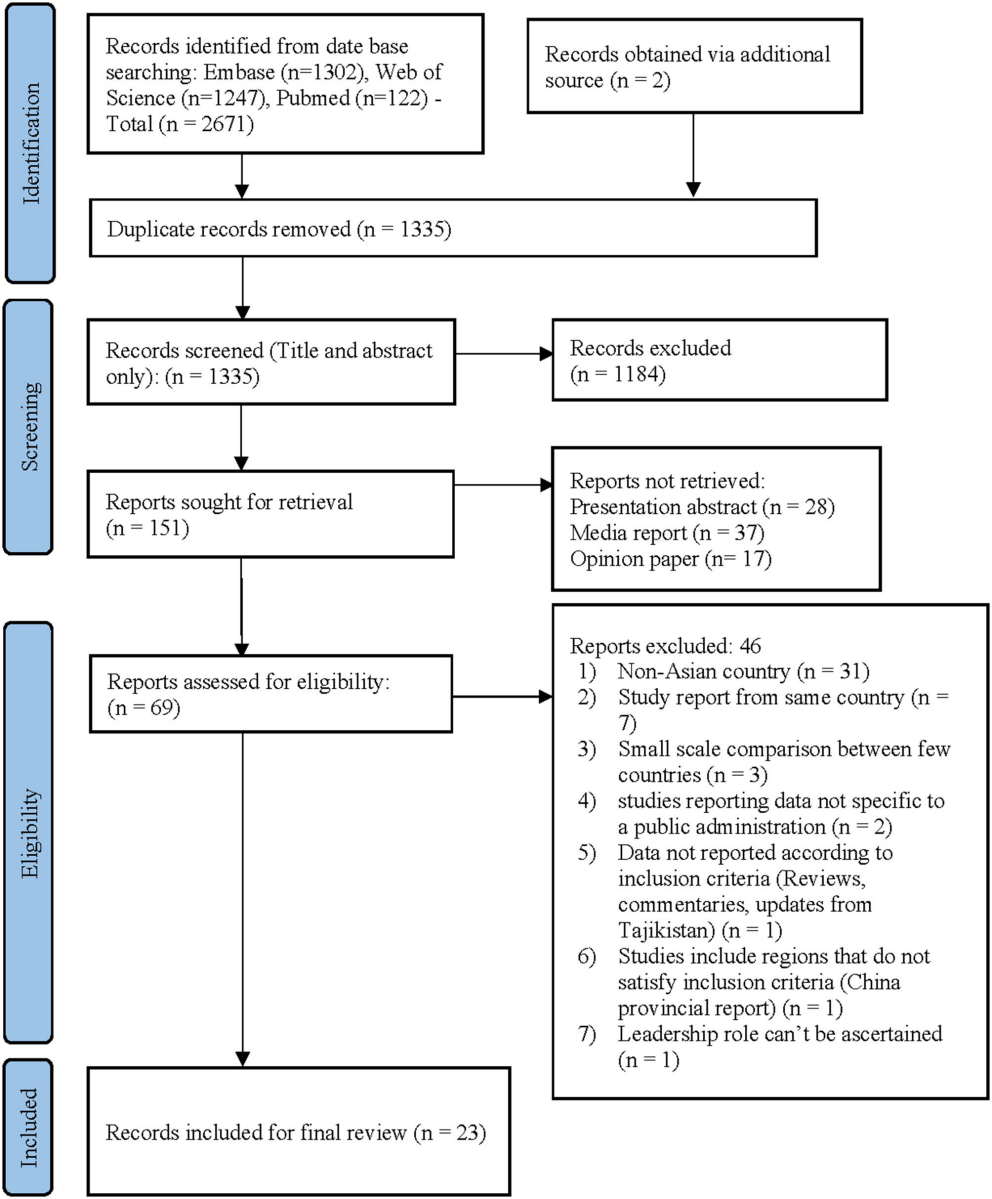
PRISMA Flow diagram. PRISMA chart illustrating our process of obtaining the 23 included articles.

Our systematic search revealed a total of 2,671 articles in the initial search. Two papers were retrieved from alternative sources after re-review. With duplicates removal, 1,335 articles remained for review. Based on title and abstract scrutiny, irrelevant publications that did not satisfy our inclusion criteria were not considered, leaving 151 articles for full-text review. Following the full-text assessment of these articles, 23 manuscripts remained for final review (Figure 1). Eight PSI have been identified within the included series (Table 2).

**Table 2:** Characteristics of the included studies with key policy enforcement and cornerstones of COVID-19 management.

**References**	**Year**	**Country**	**Journal/Publisher**	**Key policy enforcement and cornerstone**
Alam (26)	Aug 2020	Bangladesh	International Journal of Public Leadership	Ineffective bureaucratic leadership: A dysfunctionality in the COVID-19 management system is reported.
Wong et al. (27)	Jun 2020	Brunei	Journal of Global Health	Leadership within the health sector: Engagement with media influencers and local personalities paid off.
Neak et al. (28)	Jan 2021	Cambodia	Springer, Singapore	Technical/economic cooperation: Cambodia-China Free Trade Agreement signed, which aims to increase economic cooperation.
He et al. (29)^*^	May 2020	China	Policy Design and Practice	Urban grid system: The system reorganizes urban neighborhoods into a number of “grid cells”, each of which is assigned with dedicated grid controllers.
Petridou et al. (30)	Sep 2020	Cyprus	European Policy Analysis (EPA)	Lessons from the Chinese experience: Cypriot leaders indirectly drew lessons from the Chinese experience.
Hartley et al. (31)	Jun 2020	Hong Kong	Policy and Society	Community-based-political mobilization: Crisis response was unexpectedly successful due to community mobilization.
Mita Mehta et al. (32)^*^	Jul 2020	India	International Journal of Sociology and Social Policy	Netnography via online social communication: Community interpretations through online social communication showed responsible leadership being conducted in an effective way.
Djalante et al. (33)	Apr 2020	Indonesia	Progress in Disaster Science	Multi-disciplinary decision: Strengthened health-responses as outlined by WHO and proactive no-regret approach.
Rassouli et al. (34)	Sep 2020	Iran	Frontier Public Health	Managerial governance: Strongly implemented managerial concept of engaging religious leaders along with military forces and civil volunteers.
Shimizu et al. (35)	Oct 2020	Japan	Healthcare, MDPI	Health system capacity: In the early phase, Japan managed well, but weaker policy enforcement later led to the outbreak in the western Pacific region.
Lee et al. (36)^*^	Jun 2020	Republic of Korea	Policy and Society	Quadruple-loop learning: Effective interactions of backstage and frontstage policy processes.
Loi et al. (37)	Jun 2020	Macao	International Journal of Hospitality Management	Effective bureaucratic leadership: Focusing on government interventions by adopting neo-institutional theory.
San (38)	Jan 2021	Myanmar	Springer, Singapore	Collaboration with China: Myanmar has controlled outbreak well in collaboration, but has yet to prepare for second or third wave.
Rayamajhee et al. (39)	Feb 2021	Nepal	Frontier Public Health	Ineffective bureaucratic leadership: government response so far has been insufficient.
Nawaz et al. (40)^*^	Dec 2020	Pakistan	Frontier Public Health	Smart lockdown policy: All shopping malls closed except for medical services and emergency public health response, together with adoption of a multi-sectoral approach.
Vallejo et al. (41)	Jun 2020	Philippine	Progress in Disaster Science	Political leadership: Enhanced Community Quarantine and some recommendations on how the Philippines can respond to a future pandemic crisis.
Algaissi et al. (42)	Apr 2020	Saudi Arabia	Journal of Infection and Public Health	Past experience-based governance: Unprecedented precautionary strict measures were applied using the experience learned from the MERS-CoV epidemic since 2012.
Abdullah et al. (43)^*^	Jul 2020	Singapore	American Review of Public Administration	Smart nation and digital governance: Singapore's case points to an important lesson that learning-driven coordinated strategic approaches matter for effective crisis management in the long term.
Hettiarachchi et al. (44)^*^	Oct 2020	Sri Lanka	Asian Bioethics Review	The hammer and the dance: Military enforced policy with an initial strong confinement stage (the hammer), followed by a more relaxed phase (the dance).
Huang (45)	May 2020	Taiwan	Public Administration Review	Collaborative governance: A task force (command center) launched in a timely manner to implement strategies and policies.
Marome et al. (46)^*^	Jan 2021	Thailand	International Journal of Environmental Research and Public Health	Village health volunteers (VHVs): tapping on network of individuals chosen by villagers to receive basic medical training in order to help inform and support public health in their community.
Bakir (47)	Jun 2020	Turkey	Policy and Society	Presidential bureaucracy and technology collaboration: ‘Presidential bureaucracy’ under presidential system of governance to ensure implementation without delay or being vetoed or watered down.
Ivic (48)^*^	Jul 2020	Vietnam	Asian Bioethics Review	Solidarity and care: In accordance with the ethics of care which emphasizes solidarity and responsibility.

* *Highlighted public sector innovations*.

### Risk of Bias Assessment (RoB)

**Figure 2:**
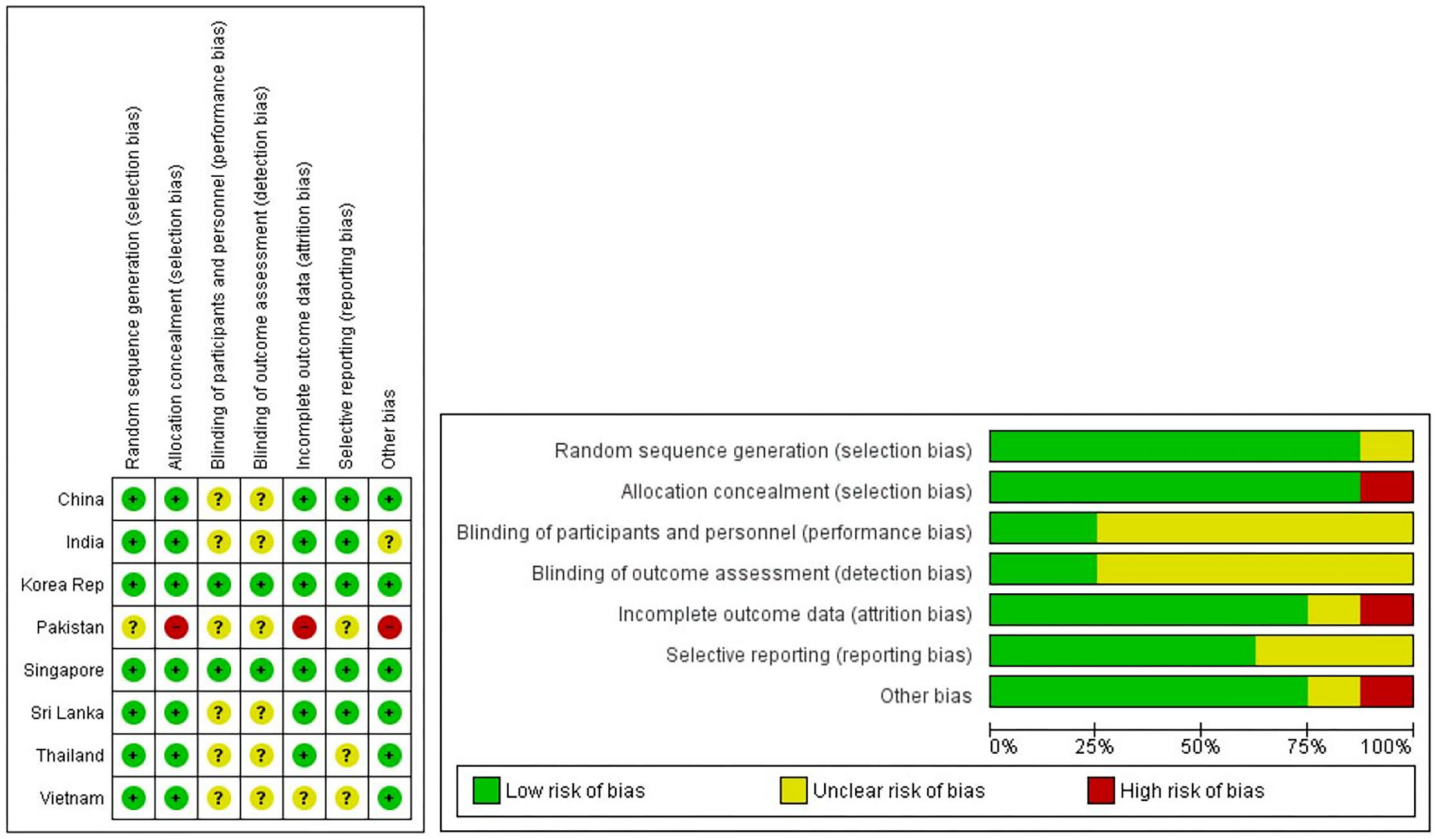
Risk of bias graphs for selected studies with PSI. This figure shows review of authors' judgements about each risk of bias item presented as percentages across all included studies. Random sequence generation were marked high due to selection bias.

The authors assessed all the included text for their risk of bias and quality of evidence by using RevMan 5.4. The risk of bias of each study was evaluated according to guidelines in chapter 8 of the Cochrane handbook of reviews (49). The overall risk of bias has been assessed for all articles, but keeping the PSI within the primary focus of the RoB has been summarized in Figure 2. As seen from Table 1 and Figure 2, the selection bias for each study was critical/substantial, which we believe can be credited to the type of study itself, the majority being observational studies and case studies describing one particular country or region. Despite this, the overall risk of bias for all the studies was classed as low/moderate. Therefore, the evidence provided by these studies was still of acceptable quality.

### Characteristics of the Included Studies

Twenty-three eligible articles (26–48) were included for final review on 31st May 2021, summarized in Table 2. As discussed earlier, all the included articles were observational studies of COVID-19 management strategies adopted by the respective governments of Asian countries. The PRISMA statement flowchart shown in Figure 1 highlights the aforementioned screening process. PRISMA chart illustrates our process of obtaining the 23 included articles. With 1,184 irrelevant records excluded based on their titles and abstracts, we reviewed the full texts of 69 articles, of which 46 were excluded, and finally, 23 articles remained for inclusion in our study. Additionally, among the included articles we identified eight papers (29, 32, 36, 40, 43, 44, 46, 48) stated “innovation” to the public sector for this disaster management.

### COVID-19 Management Scenario in Asian Countries

Asian countries are vastly diverse, both in terms of geographic distribution and population size. Despite having many dissimilarities, the COVID-19 pandemic has placed unforeseen demands on the leaders of all these nations. The potential impact of COVID-19 also has tremendous implications based on the population size. As per global population data from the 2019 Revision of World Population Prospects of U.N. shows the largest population is in China (~1.38 B) with India (~1.36 B), Indonesia (~270 M), and Bangladesh (~162 M) in top ten. Hence, the impact of having a strong and effective public administration is very much warranted. Our review shows 82.6% of the included countries had an effective public administration (Table 1) with a few exceptions, namely Bangladesh (26) and Nepal (39), which were not effective; Hong Kong (31) was relatively slow; Pakistan (40) and Saudi Arabia (42) was not known from the reports.

89.1% of the included countries had formed a national committee dedicated to COVID-19 management with multiple government authorities was directly in charge. The exceptions were India (32) and Turkey (47), where the Prime Minister and President of the respective governments were directly involved. Interestingly, there was an initial crisis denial among some countries such as Indonesia (33). The report suggests that the Indonesian government was misled by the initial “nil” incidence reported by the absence of scientific validation, after which once noticed, mitigating efforts were made through by an early presidential decree. Most of the countries reported early effective border control, Home Quarantine, or Isolation of any form. However, people did not adhere to the non-strict lockdown in Bangladesh (26), and the government was slow and reactive in the case of Hong Kong (31). Conversely, Thailand (46) was criticized by the people for an extended lockdown despite having nil community cases.

The case of Singapore (43) showed a success story by tapping on pre-existing infrastructure of “digital governance” to implement new measures such as contact tracing; in contrast, some other countries did not implement contact tracing. At the same time, non-compliance with government directives seemed to be an influential factor and has been reflected in some communities in both positive and negative ways. For example, Hong Kong (31) responded with a community-based political mobilization, while the government was reported to have a sluggish initial response. The overall effect in Asian countries is summarized in Table 3.

**Table 3:** Asian countries' responses in COVID-19 management reflecting the role of leadership.

**Country**	Population^*^	**PA system**	**Management authority**	**National Committee**	**Crisis denial**	**Early effective border control**	**Quarantine /Isolation**	**Contact tracing (DS)**	**Non-compliance**	**Political leaders**	**Religious leaders/role**	**Bureaucratic leaders**
Bangladesh (26)	162M	Non-Effective	MoHFW, DGHS, IEDCR	Yes	Yes	No	Did not adhere	Too late	Yes	Less involved	Against	Leading role
Brunei (27)	390K	Effective	NDC	Yes	No	Yes	Yes	Yes	No	Yes	-	-
Cambodia (28)	16M	Very Effective	MOH	Yes	No	Yes	Yes	Effective	No	Yes	Yes	Yes
China (29)	1.398B	Effective	NHC	Yes	Yes	-	Yes	Urban grid system	Remarkable compliance	Yes	-	Centralized leadership
Cyprus (30)	875K	Effective	Cyprus CMC	Yes	No	Yes	-	Yes	No	Yes	-	-
Hong Kong (31)	7.488m	Relatively Slow	Community-based-political mobilization	-	Yes	No	-	-	Yes, ↓ performing jurisdiction	Yes	-	No
India (32)	1.366B	Effective	PM office	-	No	Yes	Yes	-	No	Yes, PM lead	-	No
Indonesia (33)	270.6M	Effective	IAKMI	Yes	No, early decree	No	Yes, initially only from Wuhan	No	Yes	Yes, by President	Non-compliant	No
Iran (34)	82.91M	Effective	MOHME	Yes	No	-	Yes	-	Few, lack of cooperation	Yes	Yes, Supreme Leaders	No
Japan (35)	126.3M	Effective	MOH, Labor, and Welfare	Yes	Sluggish response	Yes	Yes	Yes, retrospective	Reported occasions	Weak at national level	No	Yes
South Korea (36)	51.71m	Effective	CCHQ	Yes, KCDC	Yes	No	Yes	Yes	No	Yes, led by president	No	Yes
Macao (37)	640K	Effective	Macao SAR Coordination Centre	Yes	No	Yes	Yes	-	No, highly coordinated	No	No	Yes
Myanmar (38)	54.05M	Not Known	MOHS	Yes	Yes	No	Yes	No	Not depicted	Yes	-	No
Nepal (39)	28.61M	Non-Effective	GoN	-	-	-	Yes, dangerous crowding	Inefficient	Inadequate	Yes, but corrupted	No	Not known
Pakistan (40)	216.6M	Not Known	M/O NHSRC	Yes	No	Yes	Yes, smart lockdown	Not known	Yes	Yes	Curtailed religious gatherings	Yes, central governance
Philippine (41)	108.1M	Effective	Department of Health	Yes, IATF-EID	Yes	Yes, initially selective	Yes, ECQ	Yes	-	Yes	Not known	Yes
Saudi Arabia (42)	32.27m	Not Known	Saudi Ministry of Health	Yes	No	Yes	Yes	Yes	No	Not known	Strong role	Yes
Singapore (43)	5.704M	Very Effective	Multi-Ministry Taskforce	Yes	No	Yes	Yes	Yes	No	Yes	No	Yes, strong
Sri Lanka (44)	21.8m	Effective	MOH Epidemiology Unit	Yes, Task force	No	Yes	Yes, strict curfew	Yes	Some violations	Yes, by President	Yes, confined to households	Yes, Military led
Taiwan (45)	23.816M	Very Effective	MOH and Welfare	Yes, NHCC	No	Yes	Yes	Yes, cell phone tracking	No, high public compliance	Not known	No	Yes, via Central Command
Thailand (46)	69.63M	Effective and Robust	Ministry of Public Health	Yes	No	Yes	Yes	Yes, privacy issues	-	Yes, via VHVs	No	Yes, Sendai framework
Turkey (47)	82M	Effective	Presidential office and MOH	No	No	Yes	Yes, acted early	Yes	No	Yes	No	Yes, Presidential bureaucracy
Vietnam (48)	96.46M	Effective	Vietnam's MOH	No, Solidarity and care	No, early implemented	Yes	Yes	Yes	No	Yes	No	Yes, war rhetoric of solidarity

* *Population data has been obtained from 2019 Revision of World Population Prospects, Department of Economic and Social Affairs Population Dynamics, United Nations*.

*PA, Public administration; MoHFW, Ministry of Health and Family Welfare; DGHS, Directorate General of Health Services; IEDCR, The Institute of Epidemiology Disease Control And Research; NDC, National Disaster Council; CMC, Crisis Management Center; PM, Prime Minister; NHC, China's National Health Commission; IAKMI, Indonesian Public Health Association; MOHME, Ministry of Health and Medical Education; MOHS, Ministry of Health and Sports; GoN, Government of Nepal; M/O NHSRC, Ministry of National Health Services, Regulation & Coordination; CCHQ, Central Control Headquarters; NCPCC, National Committee for Prevention and Control of COVID-19; DS, Digital surveillance; IATF-EID, Inter-agency Task Force for Emerging Infectious Diseases; ECQ, Enhanced Community Quarantine; KCDC, Korea Center for Disease Control and Prevention; NHCC, National Health Command Center; VHVs, Village health volunteers (VHVs)*.

### Effects of PSI on COVID-19 Situation

**Figure 3:**
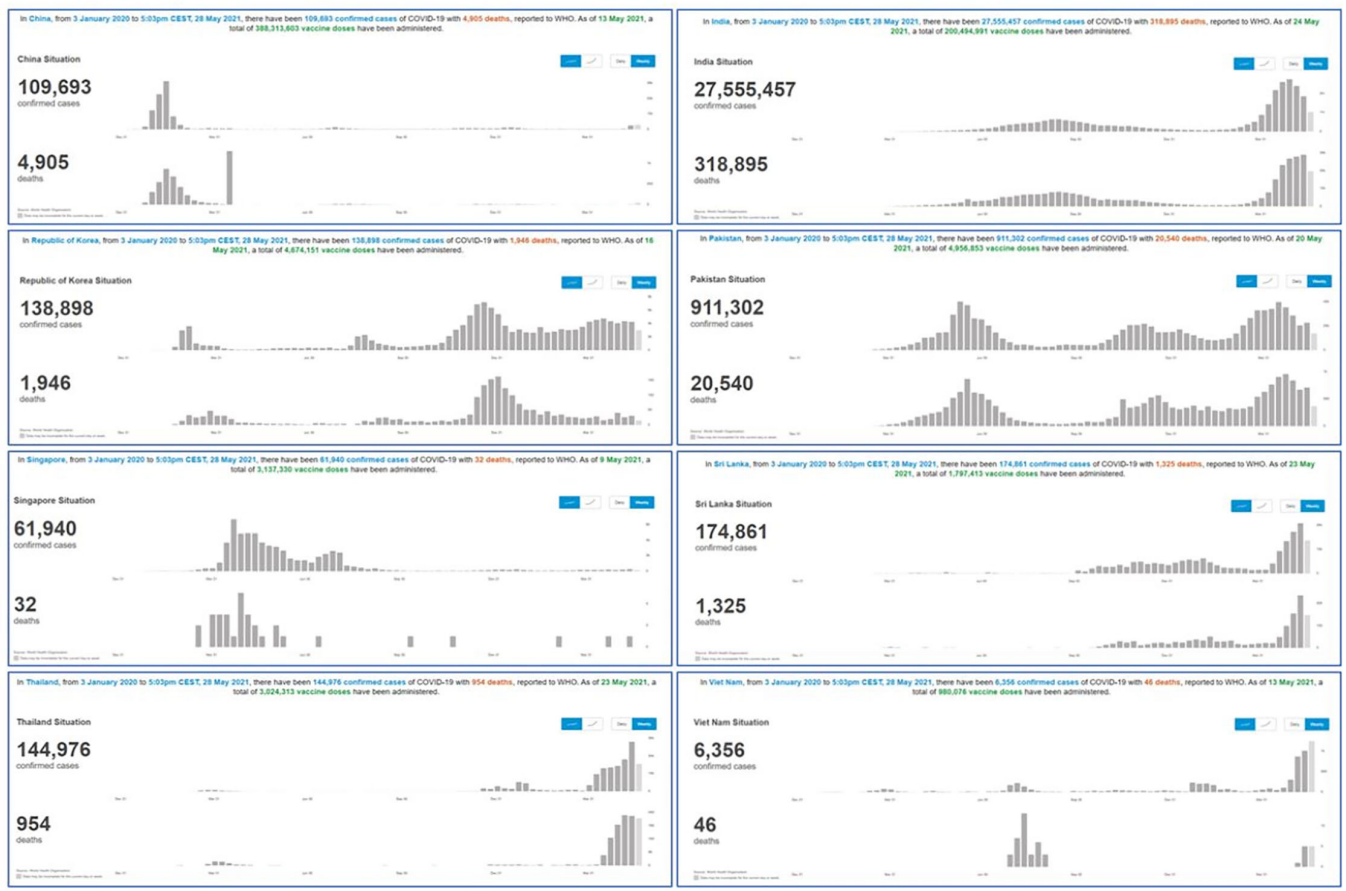
Summary of COVID-19 outcomes in PSI implemented Asian countries. WHO's dashboard of COVID-19 (50) related outcomes from January 2020 till 31st May 2021 reflecting the total number of confirmed COVID-19 cases and total number of death due to COVID-19 is Asian countries (China, India, Republic of Korea, Pakistan, Singapore, Srilanka, Thailand and Vietnam). The histogram showing the changes of outcome over the period of estimate.

Data abstracted from WHO's COVID-19 response are summarized in Figure 3 (50), showing the number of confirmed cases and the number of deaths due to COVID-19 as reported by the respective countries. In addition, a meta-analysis of the outcome was carried out using RevMan 5.4 software to assess the overall effect in selected countries with PSI (29, 32, 36, 40, 43, 44, 46, 48).

**Figure 4:**
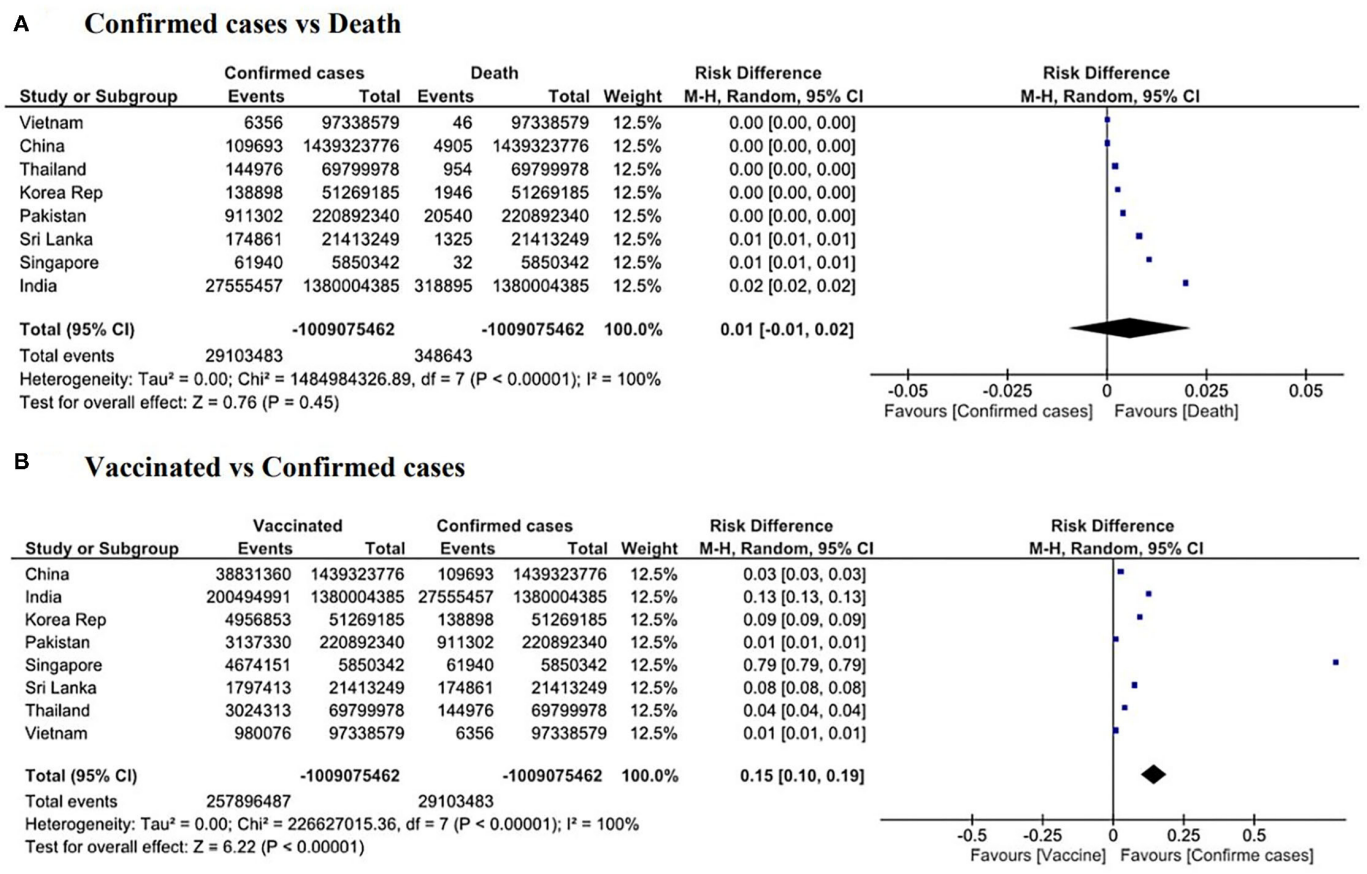
Meta-analysis of COVID-19 outcome in PSI implemented countries. Forest plots showing **(A)** Total confirmed cases vs. total number of death due to COVID-19. There is significant overall higher death in this cohort, **(B)** Overall significant number of confirmed cases when compared to vaccinated population in PSI implemented Asian countries.

The weighted mean comparison in the Cochran-Mantel-Haenszel model in random sequence generation showed that the total number of deaths in PSI implemented countries was still significantly higher when compared with a total number of confirmed COVID-19 cases (Figure 4A). However, the statistical heterogeneity of the data across countries was high. Therefore, the results of this outcome need to be interpreted with caution, with further exploration of the exact cause of heterogeneity.

Forest plot for vaccinated population compared with the total number of confirmed cases in the PSI implemented Asian countries also suggests the overall weighted mean is not in favor of the vaccination program in these countries (Figure 4B). As heterogeneity is persistent, and the *I*^2^ index is significantly higher, these results may be due to multifactorial data variation, and two or more subgroups of studies can also cause this high heterogeneity to have a different actual effect.

## Discussion

The study of the eight countries with leadership in PSI being cited revealed a range of different types of innovations (Table 2). The strategic response was categorized as a PSI as long as the innovation was new to the organization or country (4, 5, 19). Hence, for example, contact tracing apps could be considered an innovation in Thailand because this is a novel approach, which was then used to leverage its existing network of Village Health Volunteers (VHVs). Similarly, the approach adopted in Vietnam of tackling the pandemic using the language of war and emphasizing the patriotic duty of citizens to adhere to the strict lockdown was a novel strategy while at the same time leveraging the country's experience of collaboration and solidarity in the face of war.

Undoubtedly, this global pandemic is hard to control, and even though many measures have been taken, overall success is yet to be achieved. However, the leadership of some countries made little effort at the initial stage of the COVID-19 outbreak, whether by downplaying the severity of the pandemic, disregarding the importance of having a national committee, or by not having early effective border control or other safety measures. Eight Asian countries had adopted PSI to combat the global pandemic, namely China (29), India (32), Republic of Korea (36), Pakistan (40), Singapore (43), Srilanka (44), Thailand (46), and Vietnam (48). All these countries' political leaders were directly involved in the decision-making process. While the overall outcome in terms of cases and deaths is not yet satisfactory as the outbreak is still ongoing and evolving, interestingly, citizens' response varies, based on the leadership's response in managing the crisis.

### Role of Leadership (The Good, Bad, and Ugly)

Among the included countries, it could be observed that the countries that exercised only political leadership (32, 33) or weak political leadership, faced greater challenges in managing their respective outbreaks. India, for example, adopted a strong transformational leadership style, whereby the leadership aims to inspire a shared vision resulting in innovative and intrinsically motivated followers or subordinates (21). In India's case, the Prime Minister personally led all major communications and came to be seen as the face of the administration's efforts. However, the weak technocratic infrastructure and excessive bureaucratic obstacles in implementing swift measures stymied India's initial progress. Elsewhere, countries like Bangladesh (26) and Japan (35) faced challenges in adopting practical measures in the face of absent, weak, or belated political leadership.

Bureaucratic leadership appeared to have some early success in managing the outbreak. For example, the presidential bureaucracy in Turkey (47) was a known factor behind the country's early pandemic control (47), while a military-led bureaucratic enforcement in Sri Lanka (44) also led to initial success. Both countries, however, suffered a later loss of control over the pandemic. In contrast, countries such as China (29), the Republic of Korea (36), and Singapore (43), where the political leadership was combined with strong technocracy or bureaucracy, saw more significant and sustained success in controlling their outbreaks. Such countries were able to combine a strong political leadership presence with a highly technocratic style and leverage a highly-skilled bureaucracy in the public sector to implement the evolving strategies. To varying degrees, horizontal leadership can be seen at play, in Singapore and the Republic of Korea, with strong collaboration between expert groups and the public sector and allowing the problem or task to define the team. For example, in the Republic of Korea, some of the most innovative responses (such as drive-through testing centers) arose from input or feedback from expert groups or citizens, ultimately allowing the country to be one of the few that have successfully managed their outbreak without imposing national lockdowns.

At the time of writing, the leadership of countries such as Singapore (43) and Vietnam (48) were able to win the confidence of their citizens. In contrast, Indian leaders were blamed for the last surge of the pandemic (51), as they engaged in packed outdoor rallies for election campaigns and allowed religious festivals to be held with millions of attendees (51). It is evident that consistent and sustained messaging by the leadership regarding pandemic management strategies plays a key role in sustaining public confidence. In India's case, the challenges of implementing management strategies are made more complex by several factors. Firstly, the immense and diverse population places constraints on implementation and consistency of approach across all states. In contrast, countries like Singapore and the Republic of Korea are better able to maintain a consistent approach with their relatively smaller population sizes (about 6 million in Singapore and 51 million in the Republic of Korea). Secondly, there is a relatively lower level of public trust in the quality of governance in India, when compared to countries like Singapore and the Republic of Korea. These three countries' relative performance has been reflected in the annual Worldwide Governance Indicators issued by the World Bank (52). Lower levels of public trust in governance can undermine efforts to manage novel or unprecedented situations such as the COVID-19 pandemic.

In terms of religious leadership, the religious leaders from Iran, Saudi Arabia, and Sri Lanka (34, 42, 44) supported the COVID-19 regulations adopted by their respective governments. Saudi Arabia (42) deferred the Haj pilgrimage and set other historical exceptions, while Sri Lankan religious leaders endorsed the government's household confinement measures (44). In contrast, where the spiritual leadership was at odds with the administration (as in Indonesia), the mixed messaging and contradictory signals compounded the government's challenges in managing the outbreak. Elsewhere, the religious leaders in Bangladesh were against the COVID-19 measures (26), and the Pakistan government allowed religious gatherings (40), both of which seemed detrimental for managing the outbreak.

Overall, it can be seen that leadership styles can influence the level of innovation adopted and the success or failure of the strategic response. More research in this area, particularly on religious leadership as a source of innovation, could be helpful. Although many existing studies analyzed the impact of various sources of innovation, such as politicians, senior executives, supervisors, employees, citizens, industry stakeholders, or universities (7–9, 53, 54), it is not known how religious leadership functions as a source of innovation and how spiritual leadership impacts different organizational (e.g., performance) and national outcomes (e.g., economic development). Thus, this study provides significant contributions to the literature on leadership and PSI while demonstrating that top-down leadership is common and effective and religious leaders can also affect policy implementation and success at the national level.

### Role of PSIs in COVID-19 Management

A number of Asian countries were found to have adopted and implemented PSI for COVID-19 management. In China, the urban grid system (29) reflects a command structure of bureaucratic mobilization. Many aspects of crisis governance are unique and found have embedded in Chinese socio-economic culture. The netnographic study of the Indian community evaluated the prime minister's leadership and the “Janata curfew” (a form of social confinement), showing that the leadership had “relational intelligence” as they aimed to echo a similar style and voice to have a better control via the central command (32). However, after some initial success, the central leadership reported the control measures as “badly-mishandled” (51).

The quadruple-loop learning model was used to explain how the Korean government could effectively tame COVID-19 in the initial stage (36). The study concluded that the Korean government successfully responded to the COVID-19 crisis based on organizational learning theory, despite the fact that the clinical outcomes were non-resonating at the later stage of the pandemic. Interestingly, Pakistan adopted a “Smart lockdown” policy to mitigate the COVID-19 spread (50), but were never been able to bring down the number of confirmed cases and deaths. The policy's failings were rooted in poor socio-economic considerations, and government effort ultimately appeared to be ineffective (55). Nevertheless, Pakistan managed to take practical steps in the vaccination program and develop a native solution, “Pak-Vac,” with the help of China (56).

Elsewhere, multiple global authorities have lauded Singapore's success story, and the government's management strategies have been described in many publications (57). The government, having a bureaucratic culture, coordinated strategic approaches tapping on the earlier groundwork of building a smart nation with adopted policy (43). Meanwhile, in Sri Lanka, the Hammer and the Dance policy was initially enforced strictly by the military and police, leading to early and initial success; later, however, the “Dance” phase appeared to be mismatched (44), leading to rising cases. Village health volunteers (VHVs) in Thailand (46) and Solidary & Care plan by Vietnam (48) showed a similar pattern.

In most countries studied, it would appear that the initial success rate in terms of COVID-19 related confirmed cases, deaths, and vaccinations could not be sustained at the point of writing. The differentiation between countries whose leaders that adopted PSI-based measures, and those adopting non-PSI measures was also not always clear. For example, based on the percentage population that had vaccinations administered, countries with leadership in PSI registered between 0.1% (Vietnam) to 1.5% (Pakistan), 3.4% (Sri Lanka), 7.5% (Republic of Korea), and 34.9% (Singapore). Conversely, among countries where the studies did not cite leadership in PSI, vaccination rates ranged from 16.6% (Hong Kong) to 6.4% (Japan) and 2.3% (Myanmar). Similarly, the number of confirmed cases vs. deaths also presents inconclusive data between countries citing leadership in PSI and those that did not. For example, India, which maintained somewhat low numbers relative to population size for about 1 year, experienced escalated numbers of both categories from March 2021. Singapore too, which initially had low figures, entered an escalated phase a few months into 2020.

The relationship between leadership as a source of innovation in the public sector and the rates of deaths and vaccinations in this study is therefore inconclusive. Hence, while the answer to RQ1 may be partially affirmative, as 34.8% of the included countries adopted leadership in PSI (Table 1), the answer to RQ2 is not conclusive. Accordingly, the beneficial effects of PSI, and leadership in PSI, respectively, to manage the COVID-19 outbreak cannot be answered definitively. The reasons for this are manifold: most importantly, the ongoing nature of the COVID-19 outbreak makes it more challenging to pinpoint the most effective management strategies when management responses and trend data in the countries are continually evolving (58). Future systematic review after the outbreak stabilizes could shed further light on the overall impact of leadership in PSI to manage the response. Secondly, as a complex multifactorial crisis, it is challenging to identify a direct causal relationship between a few specific variables, such as the effects of leadership in PSI and the rates of confirmed cases vs. deaths and vaccinations.

Finally, the challenges that government leaders and public sector managers have faced during COVID-19 are enormous and complex. Both government leaders and the public sector continue to address problems mainly related to human resources management. For example, managers can create the conditions for employees to work in a safe and healthy environment while assessing who can work remotely. In other words, managers need to identify essential tasks, such as whether employees have to come to the job. In addition, organizations need to be more flexible and encourage work from home to those employees who are most at risk such as who have prior medical conditions and who are elderly. While doing so, workspaces can be redesigned to make sure that there are physical distances across employees and there is sufficient fresh air. Overall, what matters most for organizations is employees' well-being (9, 59–62). Investigating the effects of COVID-19 on organizations and employees, Raghavan et al. (63) find that telework and digital adoptions are two trends in COVID-19. They provide four recommendations to organizations: “improve remote work infrastructure for employees,” “use digital tools to improve communication and workflow within organizations,” “Deploy additional resources at the organizational level for digital transformation,” and “Collaborate with technology firms and academic institutions to enhance the digital skills of employees and overcome the digital divide.” [pp.11–12]. Thus, government leaders and public managers can also implement these recommendations.

Last but not least, the statistical calculation may not reflect the impact of PSI and leadership role in the short term, and long-term impact is yet to be achieved, although it has shown the initiatives taken by the leaders, perspectives of governance, and measures for COVID-19 management. Early recognition of the problem, early effective border control, avoiding crisis denial at the early stage, leveraging existing experience, digital access to information, and ensuring compliance to the government directives are found to have a strong influence in pandemic management.

### Limitations and Future Areas of Study

Firstly, the systematic review is confined to studies published on Asian countries' management of the COVID-19 outbreak. Further research into a systematic review of global reflections on the role of leadership in PSI to manage the epidemic would be helpful. In particular, as the outbreak spread rapidly across Europe and North America, further research in this area would add to the understanding of the role and impact of leadership in PSI adopted to manage the COVID-19 outbreak. Secondly, the findings of this study are preliminary due to the ongoing nature of the epidemic, where management responses and trend data in the countries are still emerging or evolving. A future systematic review after the episode could shed further light on the overall impact of leadership in PSI to manage the response. Thirdly, this study has explicitly focused on the context of the COVID-19 outbreak. An area for further research could include other contexts of turbulent problems to deepen understanding of the impact of leadership in PSI in response to such issues. Another major limitation of the current study is that PSI is not adopted in all Asian countries, limiting adequate statistical calculation. A further comparison of countries that implemented PSI, and countries that did not, could add rational values but was beyond the scope of this manuscript. Finally, the meta-synthesis did not appear significant in terms of COVID-19 management and reflected short-term outcomes. A long-term impact of the leadership role and identified PSI in the respective countries would be a potential subject for future research.

## Conclusions

The COVID-19 outbreak is still evolving, and the current review provides a cross-section of the ongoing nature of the pandemic, where management responses and trend data in the countries are still emerging or evolving. Public sector innovation applied by a few Asian countries faced mixed reactions, and overall success is yet to be achieved. Nevertheless, a decisive leadership role, early recognition of the problem, early effective border control, avoiding crisis denial at the early stage, leveraging existing experience, digital access to information, and ensuring compliance to government directives are the cornerstones to successful management of the crisis.

## Data Availability Statement

The original contributions presented in the study are included in the article/supplementary material, further inquiries can be directed to the corresponding authors.

## Author Contributions

FS and VR: conceptualization, validation, formal analysis, resources, data curation, and writing—original draft preparation. FS, VR, and MD: methodology. FS: software, visualization, and project administration. VR and MD: writing—review and editing. MD: supervision. All authors have read and agreed to the published version of the manuscript.

## Conflict of Interest

The authors declare that the research was conducted in the absence of any commercial or financial relationships that could be construed as a potential conflict of interest.

## Publisher's Note

All claims expressed in this article are solely those of the authors and do not necessarily represent those of their affiliated organizations, or those of the publisher, the editors and the reviewers. Any product that may be evaluated in this article, or claim that may be made by its manufacturer, is not guaranteed or endorsed by the publisher.

## Abbreviations

COVID-19: Corona Virus Disease 2019
PSI: Public Sector Innovation
SARS: Severe Acute Respiratory Syndrome
VUCA, Volatile, Uncertain, Complex: Ambiguous
RQ: Research Question
PRISMA: Preferred Reporting Items for Systematic Reviews and Meta-analyses
QOE: Quality of Evidence
RR: Risk Ratio
MD: Mean Difference
ROB: Risk of Bias
UN: United Nations
WHO: World Health Organization
VHV: Village Health Volunteers.
